# Bayesian active learning with model selection for spectral experiments

**DOI:** 10.1038/s41598-024-54329-w

**Published:** 2024-02-14

**Authors:** Tomohiro Nabika, Kenji Nagata, Masaichiro Mizumaki, Shun Katakami, Masato Okada

**Affiliations:** 1 Graduate School of Frontier Sciences, The University of Tokyo, Kashiwa, Chiba, 277-8561 Japan; 2 Research and Services Division of Materials Data and Integrated System, National Institute for Materials Science, Tsukuba, Ibaraki, 305-0047 Japan; 3 Faculty of Science, Course for Physical Sciences, Kumamoto University, Kumamoto, Japan

**Keywords:** Scientific data, Physics

## Abstract

Active learning is a common approach to improve the efficiency of spectral experiments. Model selection from the candidates and parameter estimation are often required in the analysis of spectral experiments. Therefore, we proposed an active learning with model selection method using multiple parametric models as learning models. Important points for model selection and its parameter estimation were actively measured using Bayesian posterior distribution. The present study demonstrated the effectiveness of our proposed method for spectral deconvolution and Hamiltonian selection in X-ray photoelectron spectroscopy.

## Introduction

Experimental design to reduce the cost of experiments is a fundamental challenge from science to industry and has been extensively studied^1^. A sequential experimental design, which selects the measurement point sequentially, has been realized by active learning^2^.

In spectral experiment, two active learning methods have been primarily evaluated. One method is to use a Gaussian process regression (GPR) model as a learning model^3–8^. As this approach is model-agnostic, it can be applied to an experiment without a formulated physical model. However, its application for the parameter estimation of physical models might be a challenge^9^. Another issue is the approach for measurement noise^2^.

The other method is to fix a physical model before the experiment and use it as a learning model^10–13^. This approach is suitable for the parameter estimation of physical models, but cannot be applied to the experiment where the physical model is not fixed.

However, in the analysis of experimental data, a physical model is selected from the candidates and then its parameters are estimated. To improve the efficiency of such experiments, active learning with model selection for parametric models is required. Active learning with model selection has been separately studied in various fields such as linear regression^14^, labeling problems^15^, and kernel selection for GPR^16^. However, none of these is applicable to spectral experiments.

In this study, we propose an active learning with model selection method using multiple parametric models as learning models to improve the model selection and its parameter estimation for spectral experiments. First, the model and its parameter posterior distribution are calculated; then, they are used to select the next measurement for model selection and its parameter estimation. The posterior probabilities are approximated using the exchange Monte Carlo method^17,18^, which allows our methods to be applied to complex physical models.

The results of the present study demonstrated the effectiveness of the proposed method for spectral deconvolution and Hamiltonian selection in X-ray photoelectron spectroscopy (XPS). In the numerical experiment, our method improved the accuracy of model selection and its parameter estimation while reducing the experiment time compared with the experiment without active learning or those with active learning using GPR.

## Bayesian model selection and its parameter estimation

We consider the problem of selecting the physical model *M* from the candidates $\mathcal {M} = \{M_1, \dots , M_K\}$ and estimating its parameter $\theta _M$. Let $D = \{x_i, y_i\}_{i=1}^N$ be the data, where $x_i$ is the measurement point and $y_i$ is the observed value. If the model *M* and its parameter $\theta _M$ are given, the probability of the data *D* is given by

1 $$\begin{aligned} p(D | M, \theta _M) = \prod _{i=1}^N p(y_i | x_i, M, \theta _M), \end{aligned}$$

where the observed value $y_i$ is assumed to be independently generated.

From Bayes’ theorem, the posterior probability of model *M* and its parameter $\theta _M$ is given by

2 $$\begin{aligned}&p(M | D) = \frac{\int p(D|\theta _M,M)p(\theta _M)p(M) d \theta _M}{\sum _{M\in \mathcal {M}} \int p(D|\theta _M,M)p(\theta _M)p(M) d \theta _M}, \end{aligned}$$

3 $$\begin{aligned}&p(\theta _M|D,M) = \frac{p(D|\theta _M,M)p(\theta _M)}{\int p(D|\theta _M,M)p(\theta _M)d \theta _M}, \end{aligned}$$

where *p*(*M*) and $p(\theta _M)$ are the prior probabilities of model *M* and its parameter $\theta _M$, respectively. The numerical computation of these posterior distribution can be realized by the exchange Monte Carlo method^17,18^.

## Bayesian active learning with model selection for parametric models

**Figure 1:**
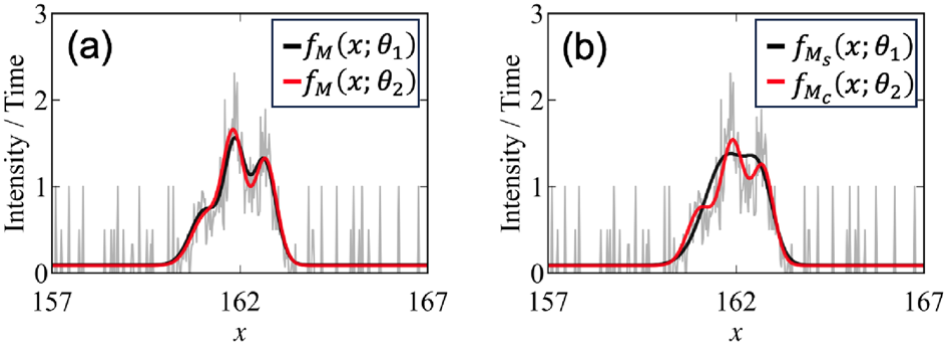
Criteria for active learning. (**a**) Parameter estimation. The gray line represents data *D* and $\theta _1, \theta _2$ follow $p(\theta _M|D,M)$. $\widetilde{\mathcal {I}}_M(x)$ corresponds to the difference between $f_M(x;\theta _1)$ and $f_M(x;\theta _2)$ integrated numerically over $p(\theta _M|D,M)$. (**b**) Model selection. The gray line represents data *D* and $\theta _1, \theta _2$ follow $p(\theta _{M_s}|D,M_s), p(\theta _{M_c}|D,M_c)$, respectively. $\widetilde{\mathcal {I}}_{s,c}(x)$ corresponds to the difference between $f_{M_s}(x;\theta _1)$ and $f_{M_c}(x;\theta _2)$ integrated numerically over $p(\theta _{M_s}|D,M_s)$ and $p(\theta _{M_c}|D,M_c)$.

The objective of the active learning is to maximize the estimation accuracy of model *M* and its parameter $\theta _M$ by sequentially selecting the next measurement point. In this study, we propose an active learning method to select the next measurement point based on two criteria: the expected improvement of the parameter estimation and that of the model selection (Fig. 1). The detailed equation transformations are given in the supplementary materials.

### Active learning criterion for parameter estimation

When $\{x,y\}$ is added to the data *D*, the information gain of the posterior distribution of the parameter $\theta _M$ is represented by

4 $$\begin{aligned} \mathcal {J}_M(x; y) = H(p(\theta _M|D,M)) - H(p(\theta _M|D\cup \{x,y\},M)). \end{aligned}$$

where *H*(*p*) is the entropy of $p(\cdot )$. Therefore, the expected gain provided by *x* is

5 $$\begin{aligned} \mathcal {I}_M(x)&= \int \mathcal {J}_M(x; y) p(y|x, D, M) d y \end{aligned}$$

6 $$\begin{aligned}&= \int _\Theta KL (p_{x,\theta _M}||p_{x,D})p_D(\theta _M)d \theta _M, \end{aligned}$$

where $p_{x,\theta }(y) = p(y|x,\theta _M,M)$, $p_D(\theta _M) = p(\theta _M|D,M)$, $p_{x,D}(y) = p(y|x,D,M) = \int p(y|x,\theta _M, M)p_D(\theta _M)d \theta$, and $KL (p||q)$ is the Kullback–Leibler (KL) divergence between *p* and *q*^19^. From convexity of KL divergence, $\mathcal {I}_M(x)$ is bounded as follows:

7 $${\mathcal{I}}_{M} (x){\text{ }} \le \int {\int K } L(p_{{x,\theta _{M} }} ||p_{{x,\theta _M' }} )p_{D} (\theta _{M} )p_{D} (\theta _M' )d\theta _M' d\theta _{M}$$

8 $$\begin{aligned}&= \widetilde{\mathcal {I}}_M(x) \end{aligned}$$

When the model *M* is expressed as

9 $$\begin{aligned} p(y|x,\theta _M,M)&= Poisson (y;f_{M}(x;\theta _M)) \end{aligned}$$

10 $$\begin{aligned}&= \frac{f_M(x;\theta _M)^y\exp (-f_M(x;\theta _M))}{y!}, \end{aligned}$$

KL divergence of $p_{x,\theta _M}$ and $p_{x,\theta _M'}$ is calculated as follows:

11 $$KL (p_{x,\theta _M}||p_{x,\theta _M'}) = f_M(x;\theta _M') - f_M(x;\theta _M) + f_M(x;\theta _M')\log \frac{f_M(x;\theta _M)}{f_M(x;\theta _M')},$$

where $f_M(x;\theta _M)$ is a physical model, and *y* follows Poisson distribution. By setting $M = \widehat{M} = argmax p(M|D)$, $\widetilde{\mathcal {I}}_M(x)$ can be calculated numerically with $p_D(\theta _M)$, which is obtained by the exchange Monte Carlo method.

Therefore, we consider selecting the next measurement point *x* that maximizes $\widetilde{\mathcal {I}}_M(x)$.

### Active learning criterion for model selection

The aforementioned criterion improves the accuracy of parameter estimation when $\widehat{M}$ is a true model. Here, we consider the criterion to make $\widehat{M}$ a true model. When data is small, a higher signal-to-noise ratio can make complex structures in spectral data less discernible, leading to a higher likelihood of selecting simpler models^20^. Therefore, we consider the criterion to select samples that favors the more complex model.

Let the second-best model $M' = argmax _{M \ne \widehat{M}}p(M|D)$, two competitive models $\{M_s, M_c\} = \{\widehat{M}, M'\}$, and $M_s$ have a smaller parameter dimension than $M_c$. (Specifically, if $\widehat{M}$ is simpler than $M'$, $M_s = \widehat{M}$ and $M_c = M'$; otherwise, $M_s = M'$ and $M_c = \widehat{M}$). We consider the following criterion to make $p(M_c|D \cup \{x,y\} )$ bigger than $p(M_s|D \cup \{x,y\} )$:

12 $$\mathcal {I}_{s,c}(x)= \int \log \frac{p(M_c|D \cup \{x,y\} )}{p(M_s|D \cup \{x,y\} )}p(y|x,D) d y$$

13 $$= \int {\log } \frac{{p(y|x,D,M_{c} )}}{{p(y|x,D,M_{s} )}}p(y|x,D,M_{c} )dy + C.{\text{ }}$$

where *C* is a constant independent of *x*.

$\frac{p(y|x,D,M_c)}{p(y|x,D,M_s)}$ is referred to as the Bayes factor, a concept well-explored in Bayesian decision theory^21,22^.

From convexity of KL divergence, $\mathcal {I}_{s,c}(x)$ is bounded as follows:

14 $$\begin{aligned}&\mathcal {I}_{s,c}(x) - C \nonumber \\&\le \int \int KL (p_{x,\theta _{M_c}}||p_{x,\theta _{M_s}})p_D(\theta _{M_c})p_D(\theta _{M_s})d \theta _{M_c}d \theta _{M_s} \end{aligned}$$

15 $$\begin{aligned}&= \widetilde{\mathcal {I}}_{s,c}(x) \end{aligned}$$

$\widetilde{\mathcal {I}}_{s,c}(x)$ can be calculated with $p_D(\theta _{M_c}),p_D(\theta _{M_s})$, which are obtained by the exchange Monte Carlo method.

Therefore, the next measurement point *x* that maximizes $\widetilde{\mathcal {I}}_{s,c}(x)$ is also selected.

## Spectral deconvolution

Our proposed method was applied to the spectral deconvolution in XPS, which poses a challenge in estimating the number of peaks and their parameters^20^.

### Problem setting

Let $M_K$ be a model with *K* peaks, the parameter set $\theta _{M_K}$ be $\theta _{M_K} = \{\{a_k, \mu _k, \sigma _k\}_{k = 1}^K ,B\}$, and the physical model $f_{M_K}(x;\theta _K)$ be $f_{M_K}(x;\theta _K) = \sum _{k = 1}^{K} a_k\exp \left( -\frac{(x-\mu _k)^2}{2\sigma _k^2}\right) + B$ (where $a_k,\mu _k,\sigma _k$, and *B* correspond to the peak intensity, peak position, peak width, and background intensity, respectively). Since the measurement is performed by photon counting in XPS, the probability distribution of the number of observed photons $p(y|f_{M}(x;\theta _M))$ is $Poisson (y;f_{M}(x;\theta _M)\times T)$ with measurement time *T*.

### Detailed algorithm for spectral deconvolution

To apply our method, a set of candidate models must be given in advance; however, in the Bayesian spectral deconvolution, the number of peaks *K* can take any integer. Therefore, we consider changing the candidate model set sequentially.

We define the initial model set as $\mathcal {M} = \{M_1,M_2,M_3\}$. At each step, let $\widehat{K}$ be the number of peaks of the best predicted model $\widehat{M}$ ($\widehat{M} = M_{\widehat{K}}$). The following model set was used in the next estimation:

16 $$\begin{aligned} \mathcal {M} = \left\{ \begin{array}{ll} \{M_1,M_2,M_3\} &{} (\widehat{K} = 1)\\ \{M_{\widehat{K} - 1},M_{\widehat{K}},M_{\widehat{K}+ 1}\}. &{} (otherwise ) \end{array} \right. \end{aligned}$$

In addition, in the spectral measurement, a short time measurement is performed first, followed by a long time measurement. The specific algorithm that takes these considerations into account is shown in Algorithm 1.

**Algorithm 1:**
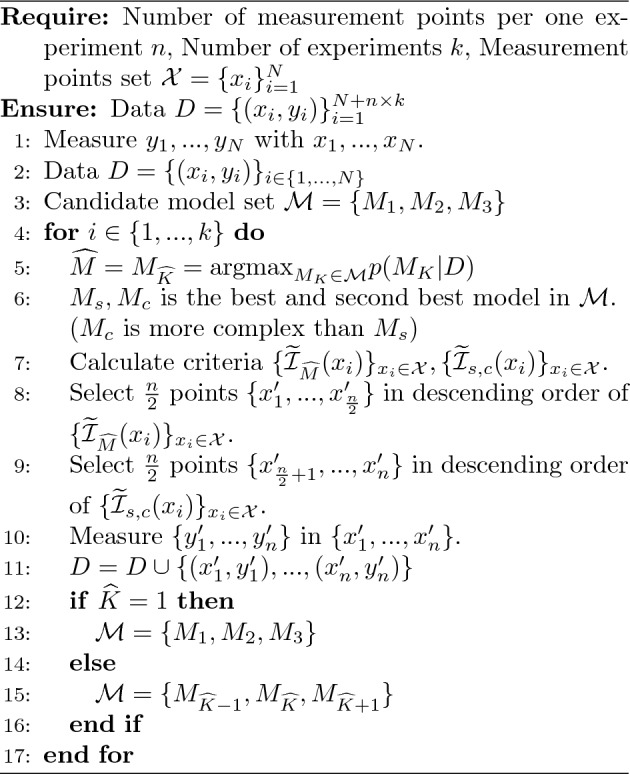
Sequential experiment for Bayesian spectral deconvolution.

### Conventional methods

We compare our method with the following two conventional methods.

#### Passive learning

Passive learning measures the same measurement time at all measurement points. This method is the most common method in spectral experiments.

#### Active learning with GPR

In active learning with GPR, a GPR model is used as a learning model, and the next measurement point that maximizes the expected improvement of the measured value estimation is selected. A detailed algorithm is given in the supplementary materials.

### Result

Let the true model be the model $M_3$ with $K=3$ peaks, and the true values of the parameters $\theta _{M_3}^* = \{ \{a_k^*,\mu _k^*,\sigma _k^*\}_{k=1}^3,B^*\}$ be as follows:

17 $$\begin{aligned} \begin{pmatrix} a_1^*\\ a_2^*\\ a_3^*\\ \end{pmatrix}&= \begin{pmatrix} 0. 587\\ 1.522\\ 1.183\\ \end{pmatrix} ,\ \begin{pmatrix} \mu _1^*\\ \mu _2^*\\ \mu _3^*\\ \end{pmatrix} = \begin{pmatrix} 161.032\\ 161.852\\ 162.677\\ \end{pmatrix} , \end{aligned}$$

18 $$\begin{aligned} \begin{pmatrix} \sigma _1^*\\ \sigma _2^*\\ \sigma _3^*\\ \end{pmatrix}&= \begin{pmatrix} 0.341\\ 0.275\\ 0.260\\ \end{pmatrix},\ B = 0.1. \end{aligned}$$

**Figure 2:**
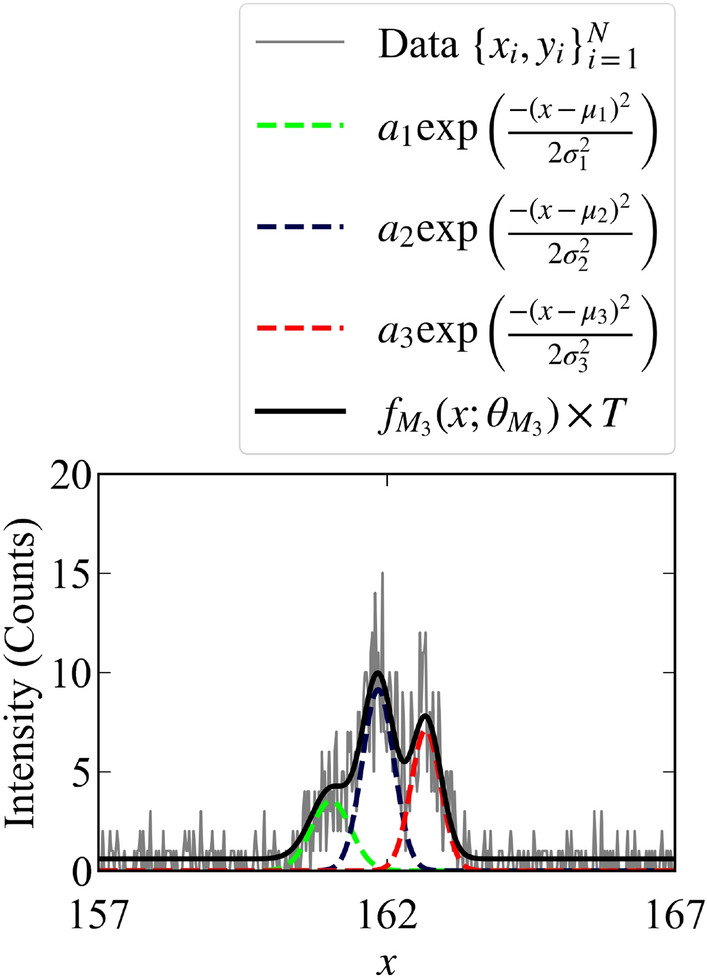
Value of the modeling function $f_{M_3}(x;\theta _{M_3})$ and the example of the observed data $\{x_i,y_i\}_{i=1}^{400}$ when $T = 6$.

The modeling function $f_{M_3}(x;\theta _{M_3}^*)$ is shown in Fig. 2.

Let the measurement time for one measurement in active learning *T* be $T=1$, number of measurement points per one experiment $n = 10$, and the candidate set of measurement points $\mathcal {X}$ be $\mathcal {X} = \{157 + 0.025(i-1)\ (eV) \}_{i=1}^{400}$. The prior distributions are shown in the supplementary materials.

**Figure 3:**
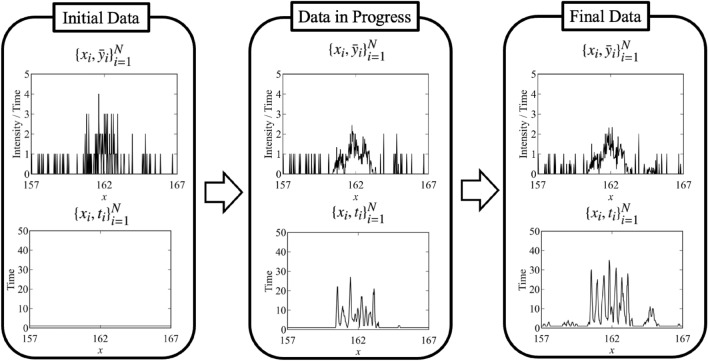
Flow of the proposed method for spectral deconvolution. The upper figure shows the observed values per measurement time $\bar{y}_i = \frac{\sum _{x_j = x_i} y_j}{t_i}$, and the lower figure shows the total measurement time per measurement point $t_i = \#\{j|x_j = x_i\}\times T$. Although the signal-to-noise ratio of the initial data is poor at all measurement points, the signal-to-noise ratio of the data near the peak is improved by repeating the experiments.

**Figure 4:**
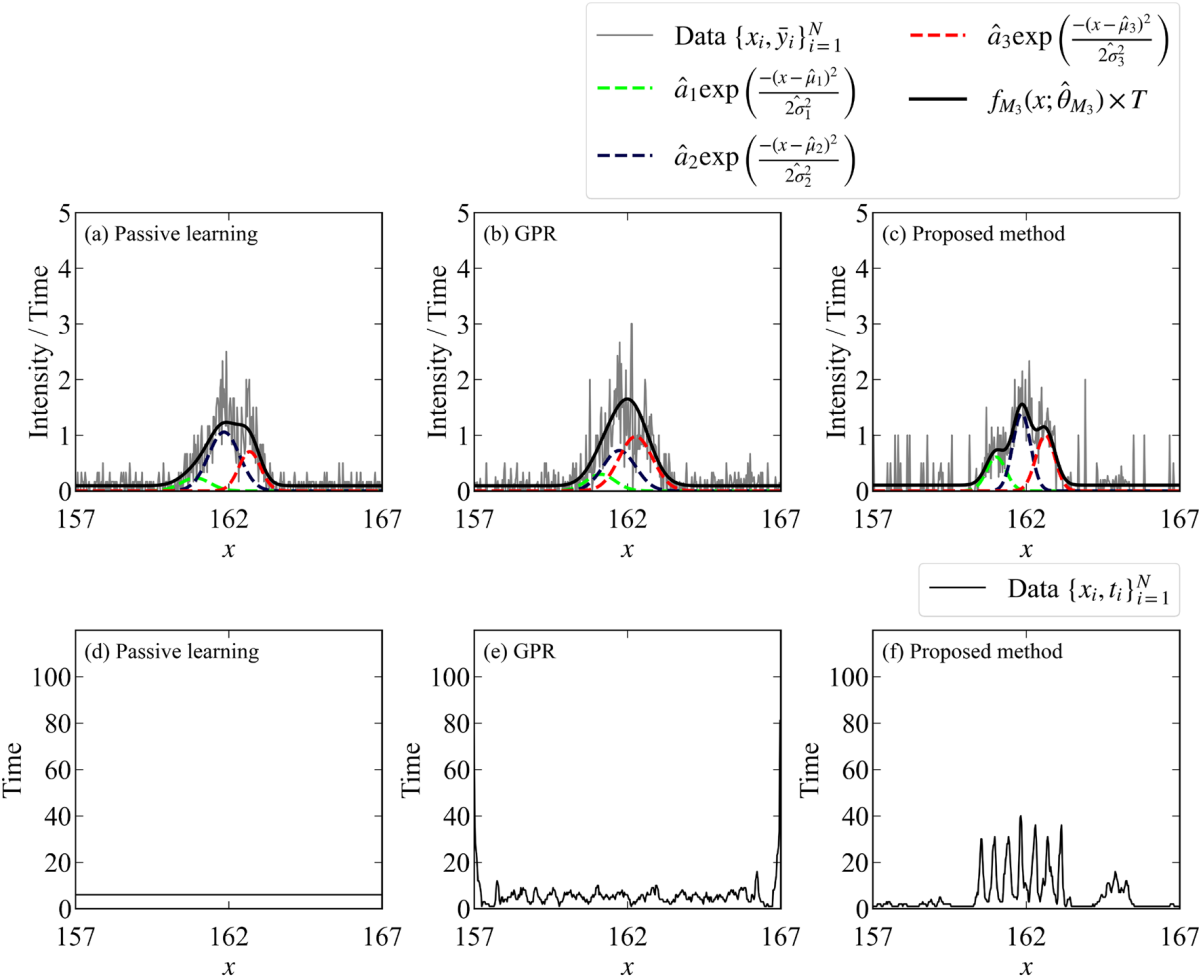
Data and fitting obtained by experiments on the spectral deconvolution. The upper figure shows that the number of photons observed per measurement time $\bar{y}_i$ and the fitting by the MAP estimator. The lower figure shows the total measurement time per measurement point. (**a**,**d**) Passive learning. (**b**,**e**) Active learning with GPR. (**c**,**f**) Proposed method.

The flow of the measurement is shown in Fig. 3. The signal-to-noise ratio is poor at all points at first. However, as the experiment progresses, the signal-to-noise ratio near the peaks improves due to focused measurements. The data and the fitting by the MAP estimator ($\hat{\theta }_{M_3} = \{\{\hat{a}_k, \hat{\mu }_k, \hat{\sigma }_k\}_{k = 1}^K ,\hat{B}\} = \underset{\theta }{\text {argmax }}p(\theta | D, M_3))$ when the total measurement time is 2400 are shown in Fig. 4 (the parameter indices are set so that $\mu _1<\mu _2<\mu _3$). This figure shows that the proposed method focuses on the measurement points near the peaks that are considered to be important in the spectral deconvolution.

**Figure 5:**
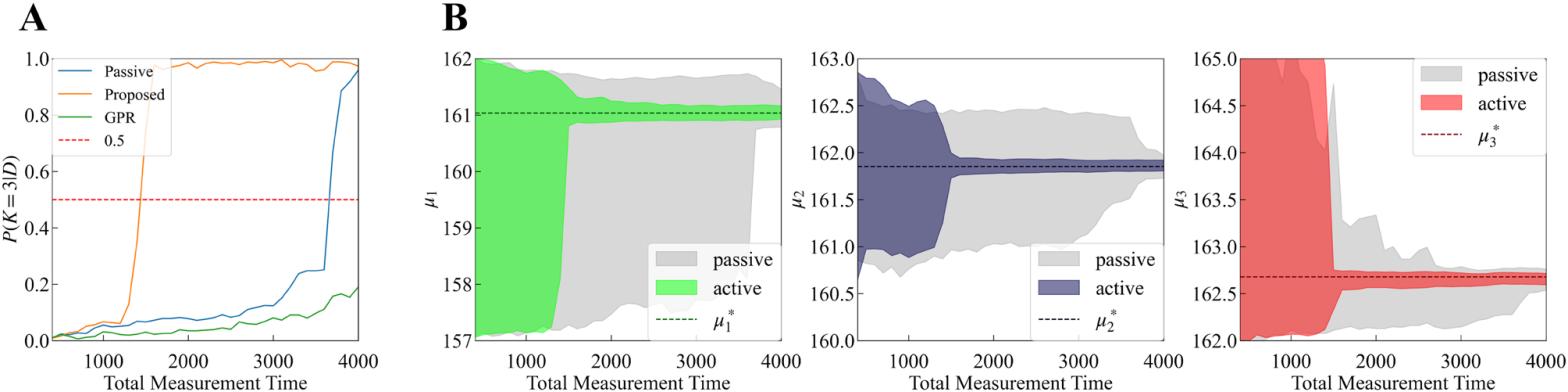
(**A**) Model selection results. The horizontal axis is the total measurement time; the vertical axis, the probability of the true model; the blue line, the result of passive learning; the green line, the result of active learning with GPR; and the orange line, the result of our method. (**B**) 99% credible interval of the parameter estimation of peak positions $\mu _1, \mu _2, \mu _3$. The horizontal axis is the total measurement time. The gray area represents the result of passive learning; the colored area, the result of our method; and the dotted lines, the true value of $\mu _1, \mu _2, \mu _3$.

In addition, we calculated $p(K = 3 | D)$ and $p(\theta _{M_3}|D, M_3)$ when the total measurement time is $\{ 400 + 100i\}_{i=0}^{36}$. Figure 5A shows the result of the model selection. Active learning with GPR does not improve the model selection because of the high intensity measurement noise. However, our method improves the model selection compared to passive learning. Figure 5B shows the 99% credible interval of the parameter estimation of peak positions $\mu _1, \mu _2, \mu _3$. Our method narrowed the interval width and improved the parameter estimation.

**Figure 6:**
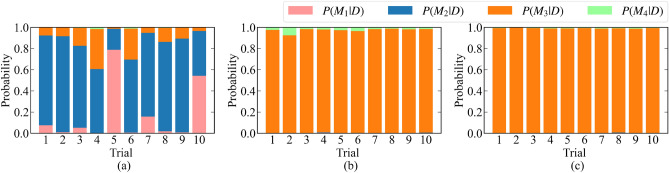
Bar graphs of $p(M_1|D),p(M_2|D),p(M_3|D),p(M_4|D)$ for the 10 independent trials. (**a**) Passive learning with a total measurement time of 2400. (**b**) Proposed method with a total measurement time of 2400. (**c**) Passive learning with a total measurement time of 7200.

**Figure 7:**
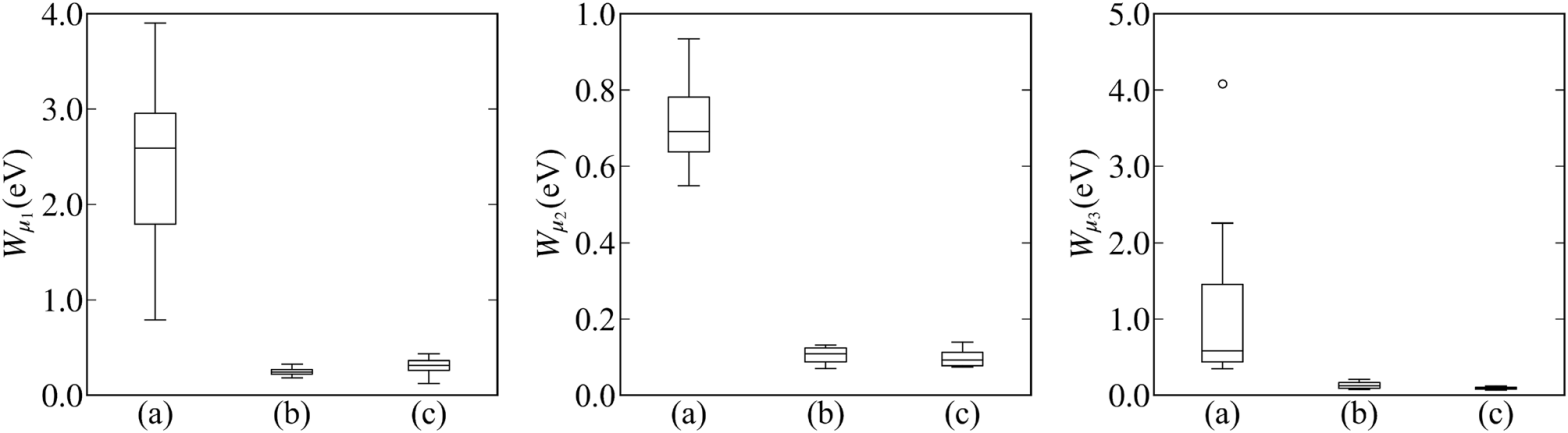
Boxplots represent the accuracy of parameter estimation of the peak positions. The left panel, middle panel, and right panels show the boxplots of $W_{\mu _1},W_{\mu _2}$, and $W_{\mu _3}$ respectively. (**a**) Passive learning with a total measurement time of 2400. (**b**) Proposed method with a total measurement time of 2400. (**c**) Passive learning with a total measurement time of 7200.

Moreover, the results of 10 independent measurements were compared: (a) passive learning with total measurement time 2400, (b) active learning with total measurement time 2400, and (c) passive learning with total measurement time 7200. Figure 6 shows the result of the model selection. Figure 7 shows the parameter estimation accuracy. Here, we defined the parameter estimation accuracy $W_{\mu _1}, W_{\mu _2}, W_{\mu _3}$ for $\mu _1,\mu _2,\mu _3$ as follows:

19 $$\begin{aligned} W_{\mu _i} = \max _{\alpha \in [0.005,0.995]}|\mu _i^* - \mu _{i,\alpha }| \end{aligned}$$

where

20 $$\begin{aligned} \mu _{i,\alpha }&= \min _{\mu } \left\{ \left( \int _{\mu _i < \mu }p(\mu _i|D,K)d \mu _i\right) > \alpha \right\} . \end{aligned}$$

Both results show that our method improved the estimation accuracy and shortened the measurement time.

## Hamiltonian selection

The Hamiltonian selection in XPS^23^ was also considered in this study.

### Problem setting

Let $M_2$ be a model using a two-state Hamiltonian, $H_2$, and $M_3$ be a model using a three-state Hamiltonian $H_3$, and let $\mathcal {M} = \{M_2,M_3\}$ be a set of candidate models. Let $\theta _{M_2} = \{\Delta , V, \Gamma , U_{fc},b\}$ and $\theta _{M_3} = \{\Delta , V, \Gamma , U_{fc},U_{ff}, b\}$. The physical model $f_{M_2}(x;\theta _{M_2})$ and $f_{M_3}(x;\theta _{M_3})$ are shown in the supplementary materials. As the measurement is performed by photon counting in XPS, the probability distribution of the number of observed photons $p(y|f_{M}(x;\theta _M))$ is considered to be $Poisson (y;f_{M}(x;\theta _M)\times T)$ with measurement time *T*.

### Detailed algorithm for Hamiltonian selection

Unlike in the case of spectral deconvolution, the model set $\mathcal {M} = \{M_2,M_3\}$ is fixed. The specific algorithm is shown in Algorithm 2.

**Algorithm 2:**
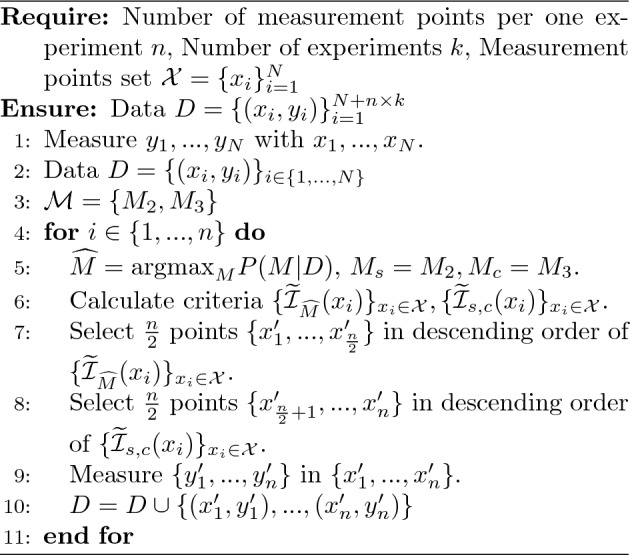
Sequential experiment for Bayesian Hamiltonian Selection.

### Conventional methods

We compare our method with passive learning and active learning with GPR as in the case of spectral deconvolution.

### Result

Let the true model be the model $M_3$ with $H_3$ and the true values of its parameters be as follows:

21 $$\begin{aligned} \Delta ^*&= 7.66, V^* = 0.76, U_{ff} = 10.5, \end{aligned}$$

22 $$\begin{aligned} U_{fc}&= 12.7,\ \ \Gamma = 0.7,\ \ \ \ \ b = 0. \end{aligned}$$

**Figure 8:**
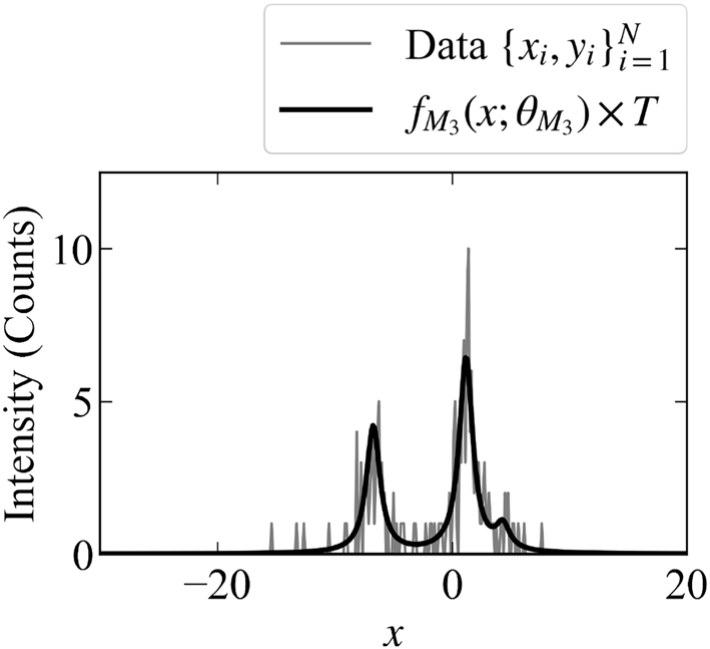
Plot of the modeling function $f_{M_3}(x;\theta _{M_3}^*)$ and the example of the observed data $\{x_i,y_i\}_{i=1}^{400}$ when $T = 25$. The peak around $x=5$ is small, indicating the difficulty of the model selection.

This true parameter is derived from^23^. The physical function $f_{M_3}(x;\theta _KL divergence o{M_3})$ with the true parameter $\theta _{M_3}^* = \{\Delta ^*, V^*, \Gamma ^*, U_{fc}^*,U_{ff}^*, b^*\}$ is shown in Fig. 8. The peak around $x=5$ is small, indicating that the model selection from model $M_2$ that generates two peaks and model $M_3$ that generates three peaks is difficult. Let the measurement time for one measurement in active learning *T* be $T=1$, number of measurement points per one experiment $n = 10$, and the candidate set of measurement points $\mathcal {X}$ be $\mathcal {X} = \{-30 + 0.125(i-1)\}_{i=1}^{400}$. The prior distribution is shown in the supplementary materials.

**Figure 9:**
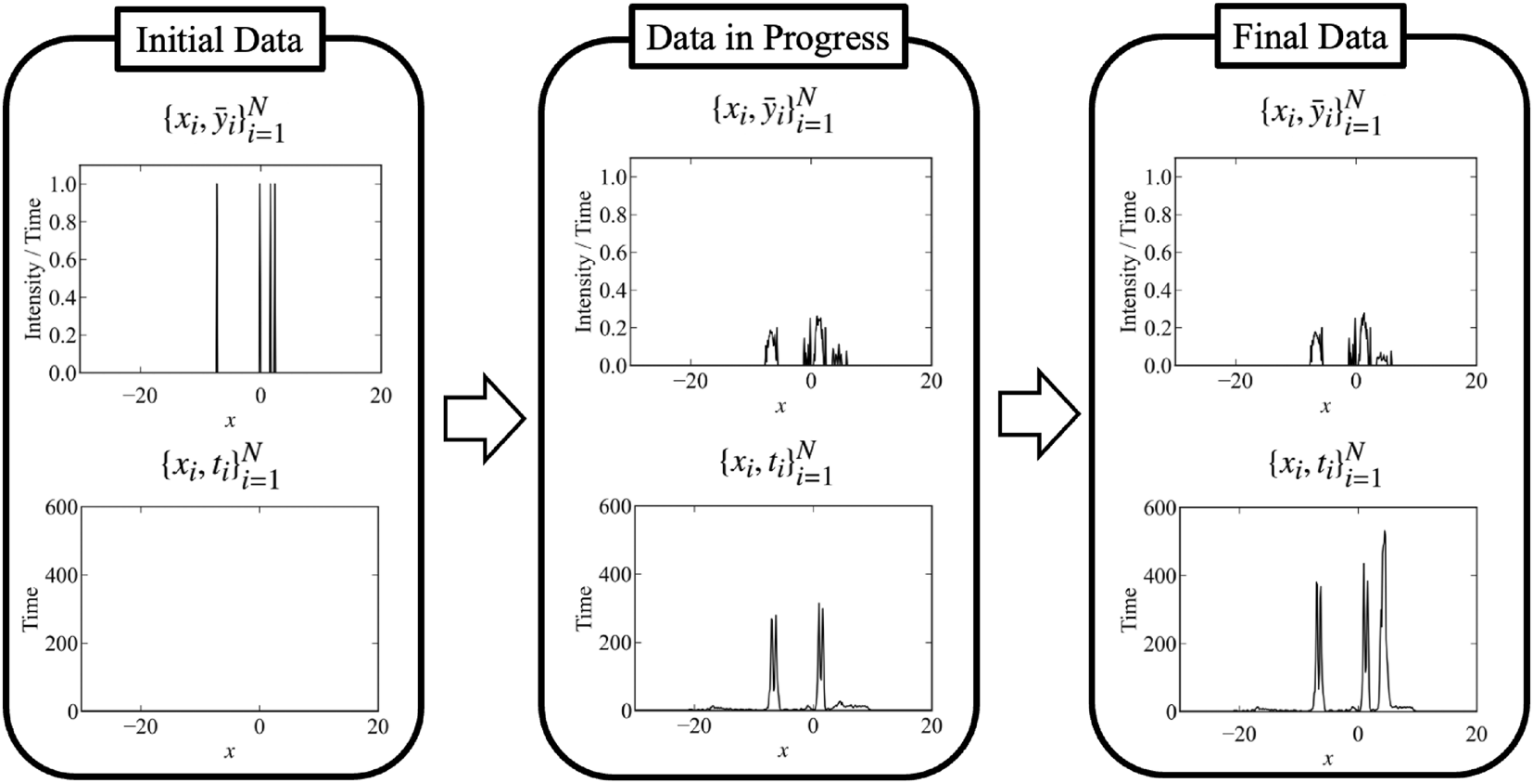
Flow of the proposed method for Hamiltonian selection. The upper figure shows the observed values per measurement time $\bar{y}_i = \frac{\sum _{x_j = x_i} y_j}{t_i}$, and the lower figure shows the total measurement time per measurement point $t_i = \#\{j|x_j = x_i\}\times T$. It can be observed that the area near the peaks, particularly near the small peak around $x=5$, is measured intensively.

**Figure 10:**
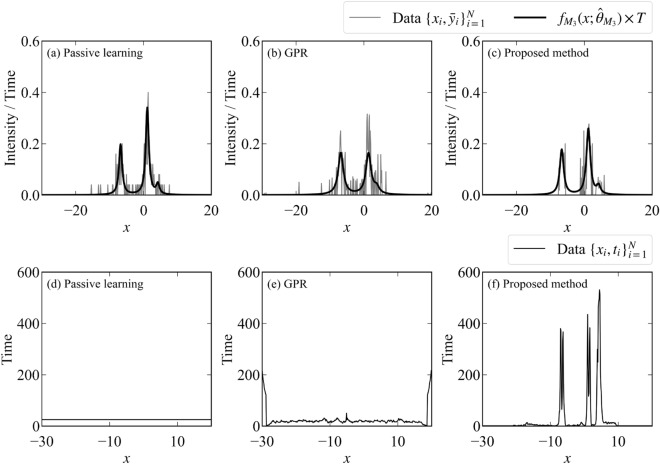
Data and fitting obtained by experiments on the Hamiltonian selection. The upper figure shows that the number of photons observed per measurement time $\bar{y}_i$ and the fitting by the MAP estimator. The lower figure shows the total measurement time per measurement point. (**a**,**d**) Passive learning. (**b**,**e**) Active Learning with GPR. (**c**,**f**) Proposed method.

The flow of the measurement is shown in Fig. 9. The signal-to-noise ratio is poor at all points at first. However, as the experiment progresses, the signal-to-noise ratio near the peaks improves due to focused measurements. The data and the fitting by the MAP estimator ($\hat{\theta }_{M_3} = \underset{\theta }{\text {argmax }}p(\theta | D, M_3))$ when the total measurement time is 10,000 are shown in Fig. 10. This figure shows that the proposed method focuses on the area near the peaks, particularly near the small peak around $x=5$.

**Figure 11:**
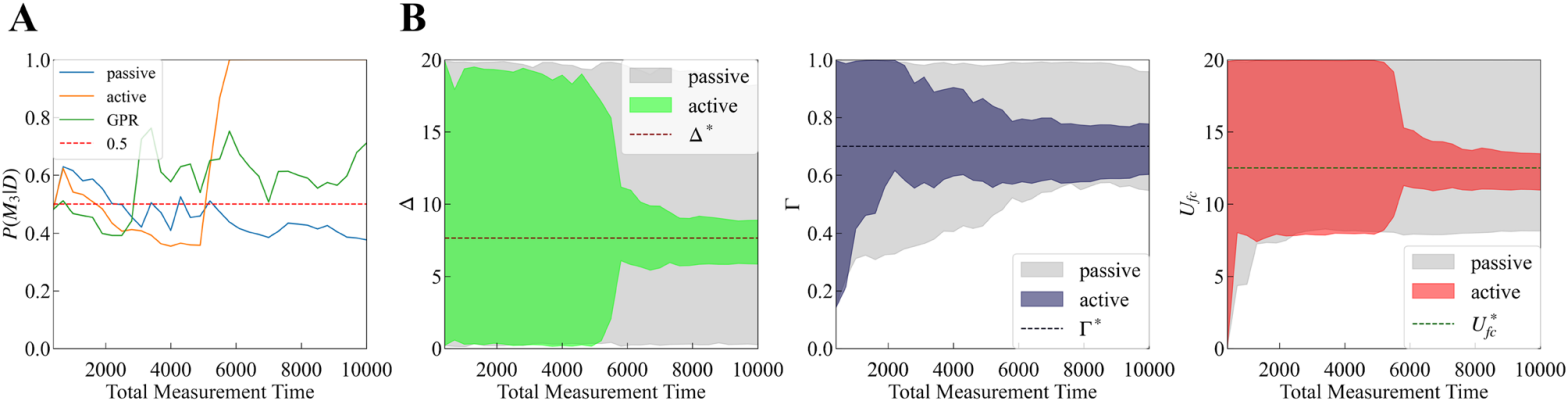
(**A**) Model selection results. The horizontal axis is the total measurement time; the vertical axis is the probability of the true model; the blue line is the result of passive learning; the green line is the result of active learning with GPR; and the orange line is the result of our method. (**B**) 99% credible interval of the parameter estimation of $\Delta , \Gamma , U_{fc}$. The horizontal axis is the total measurement time. The gray area indicates the result of passive learning, while the colored area indicates the result of our method; the dotted lines represent the true value $\Delta , \Gamma , U_{fc}$.

In addition, we calculated $p(M_3|D)$ and $p(\theta _{M_3}|D, M_3)$ when the total measurement time is $\{ 400 + 300i\}_{i=0}^{32}$. Figure 11A shows the result of the model selection. As in the previous section, active learning with GPR did not improve the model selection because of the high intensity measurement noise. However, our method improved the model selection compared to passive learning. Figure 11B shows the 99% credible interval of the parameter estimation of $\Delta , \Gamma , U_{fc}$. Our method narrowed the interval width and improved the parameter estimation.

**Figure 12:**
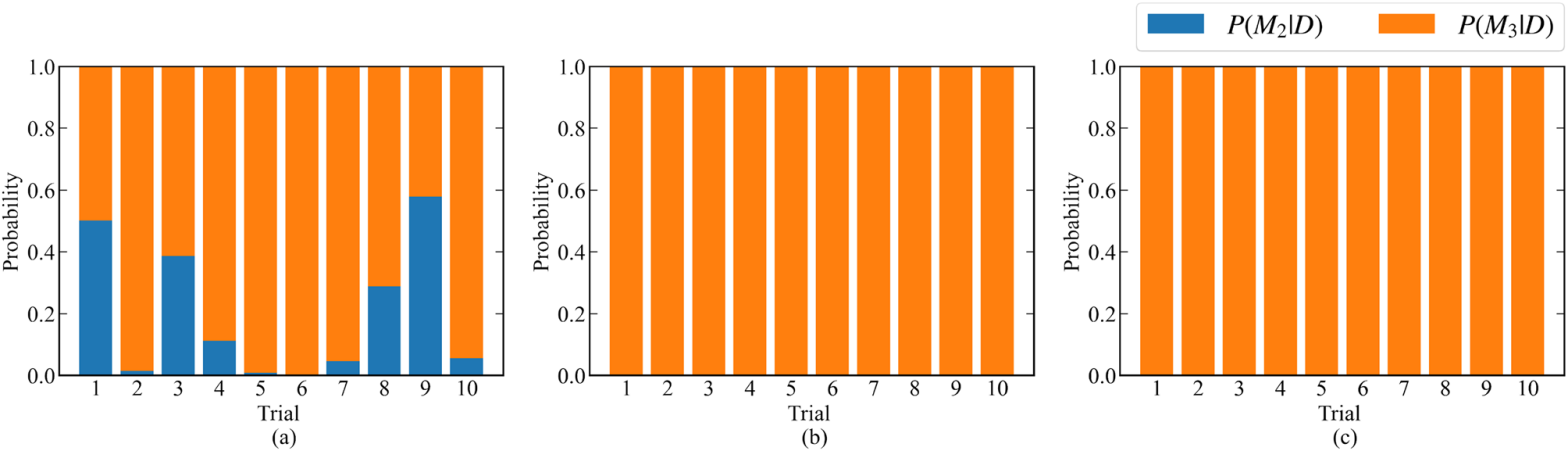
Bar graphs of $p(M_2|D),p(M_3|D)$ for the 10 independent trials. (**a**) Passive learning with a total measurement time of 10,000. (**b**) Proposed method with a total measurement time of 10,000. (**c**) Passive learning with a total measurement time of 40,000.

**Figure 13:**
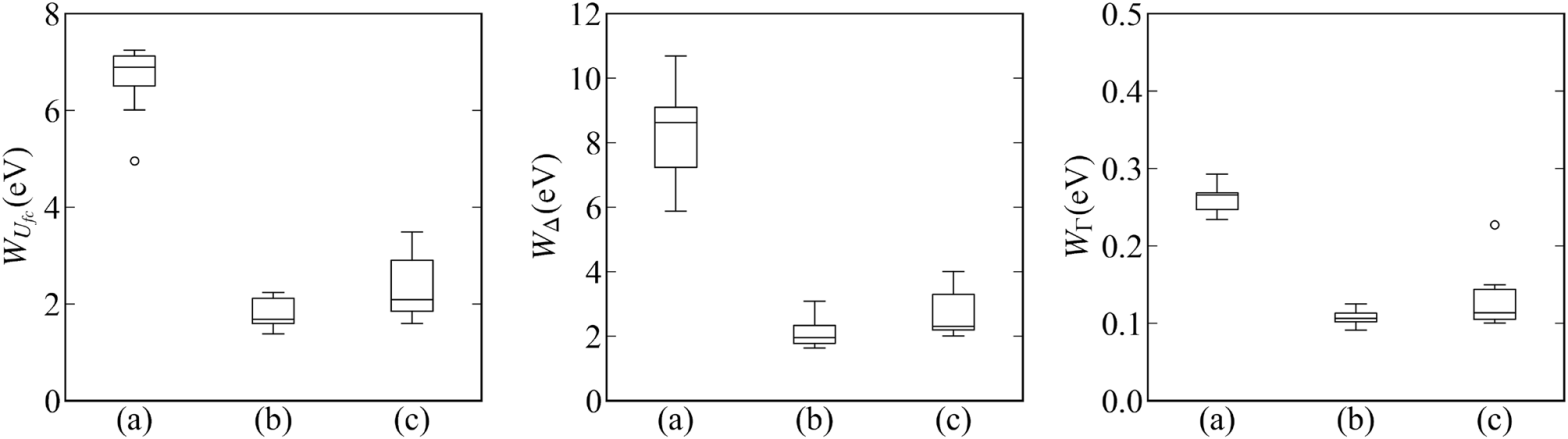
Boxplots represent the accuracy of parameter estimation of Hamiltonian parameters. The left panel, middle panel, and right panels show the boxplots of $W_{\Delta },W_{\Gamma }$, and $W_{U_{fc}}$, respectively. (**a**) Passive learning with a total measurement time of 10,000. (**b**) Proposed method with a total measurement time of 10,000. (**c**) Passive learning with a total measurement time of 40,000.

Moreover, 10 independent measurements were performed, and the following results were compared: (a) passive learning with total measurement time of 10,000, (b) active learning with total measurement time of 10,000, and (c) passive learning with total measurement time of 40,000. Figure 12 shows the result of the model selection. Figure 13 shows the accuracy of parameter estimation. Here, we defined the accuracy of parameter estimation $W_{\Delta }, W_{\Gamma }, W_{U_{fc}}$ for $\Delta , \Gamma , U_{fc}$ as follows:

23 $$\begin{aligned} W_{\Delta }&= \max _{\alpha \in [0.005,0.995]}|\Delta ^* - \Delta _{\alpha }|, \end{aligned}$$

24 $$\begin{aligned} W_{\Gamma }&= \max _{\alpha \in [0.005,0.995]}|\Gamma ^* - \Gamma _{\alpha }|, \end{aligned}$$

25 $$\begin{aligned} W_{U_{fc}}&= \max _{\alpha \in [0.005,0.995]}|U_{fc}^* - U_{fc,\alpha }|, \end{aligned}$$

where

26 $$\begin{aligned} \Delta _{\alpha }&= \min _{\Delta } \left\{ \left( \int _{\Delta < \Delta ^*}p(\Delta |D,M_3)d \Delta \right) > \alpha \right\} , \end{aligned}$$

27 $$\begin{aligned} \Gamma _{\alpha }&= \min _{\Gamma } \left\{ \left( \int _{\Gamma < \Gamma ^*}p(\Gamma |D,M_3)d \Gamma \right) > \alpha \right\} , \end{aligned}$$

28 $$\begin{aligned} U_{fc,\alpha }&= \min _{U_{fc}} \left\{ \left( \int _{U_{fc} < U_{fc}^*}p(U_{fc}|D,M_3)d U_{fc}\right) > \alpha \right\} . \end{aligned}$$

Both results show that our method improved the estimation accuracy and shortened the measurement time.

## Conclusion and future work

We developed an active learning method using multiple parametric models as learning models to improve the accuracy of model selection and parameter estimation in spectral experiments. In our method, the next measurement points that are important for model selection and its parameter estimation were selected using the model and its parameter posterior distribution. We applied our method to two spectral experiments, namely spectral deconvolution and Hamiltonian selection. In both experiments, the proposed method improved the model selection and accuracy of parameter estimation compared with passive learning and active learning with GPR.

To apply our method to a broader range of actual spectral experiments, we need to consider the following points. Firstly, there is a concern about the calculation cost of the proposed method. To reduce the actual experimental time using the proposed method, the computational time of the Monte Carlo method should be sufficiently small compared to the experiment time. Nevertheless, in cases with a large number of parameters, the exploration range of the Monte Carlo method expands, leading to longer convergence times. Additionally, the computation time per iteration often scales proportionally with the number of measurement points. Therefore, in scenarios with a high number of measurement points, such as in the case of high-dimensional spectral data, the measurement time can significantly increase. To mitigate computational time, one approach is to employ the concept of sequential Monte Carlo methods^24^, utilizing samples obtained from previous simulations to perform sampling from the new posterior distribution. Moreover, the convergence can be improved by appropriately setting the prior distribution using prior knowledge about the experiment^25^.

Another challenge is adjusting the Monte Carlo parameters automatically. The Monte Carlo method has many parameters and setting them up for each experiment is difficult. Thus, an algorithm like NUTS^26^ to adjust the Monte Carlo parameters will be required.

Finally, our method is only applicable when candidate models are known in advance. However, background functions often have limited prior knowledge, making modeling challenging in many cases. Such challenges can be solved by using semi-parametric models^27^.

### Supplementary Information

Supplementary Information.

## Data Availability

The data and codes that support the findings of this study are available from the corresponding author upon request.

## References

[1] Rainforth, T., Foster, A., Ivanova, D. R. & Smith, F. B. Modern Bayesian experimental design. arXiv:2302.14545 (arXiv preprint) (2023).

[2] Hino, H. Active learning: Problem settings and recent developments. arXiv:2012.04225 (arXiv preprint) (2020).

[3] Ueno T (2018). Adaptive design of an X-ray magnetic circular dichroism spectroscopy experiment with Gaussian process modelling. NPJ Comput. Mater..

[4] Ueno T, Ishibashi H, Hino H, Ono K (2021). Automated stopping criterion for spectral measurements with active learning. NPJ Comput. Mater..

[5] Noack MM (2019). A kriging-based approach to autonomous experimentation with applications to X-ray scattering. Sci. Rep..

[6] Noack MM, Doerk GS, Li R, Fukuto M, Yager KG (2020). Advances in kriging-based autonomous X-ray scattering experiments. Sci. Rep..

[7] Noack MM (2021). Gaussian processes for autonomous data acquisition at large-scale synchrotron and neutron facilities. Nat. Rev. Phys..

[8] Holman EA (2020). Autonomous adaptive data acquisition for scanning hyperspectral imaging. Commun. Biol..

[9] Teixeira Parente M (2023). Active learning-assisted neutron spectroscopy with log-Gaussian processes. Nat. Commun..

[10] Dushenko S, Ambal K, McMichael RD (2020). Sequential Bayesian experiment design for optically detected magnetic resonance of nitrogen-vacancy centers. Phys. Rev. Appl..

[11] McMichael RD, Dushenko S, Blakley SM (2021). Sequential Bayesian experiment design for adaptive Ramsey sequence measurements. J. Appl. Phys..

[12] McMichael RD, Blakley SM (2022). Simplified algorithms for adaptive experiment design in parameter estimation. Phys. Rev. Appl..

[13] Caouette-Mansour M (2022). Robust spin relaxometry with fast adaptive Bayesian estimation. Phys. Rev. Appl..

[14] Sugiyama, M. & Rubens, N. Active learning with model selection in linear regression. In *Proceedings of the 2008 SIAM International Conference on Data Mining*, 518–529 (2008).

[15] Ali, A., Caruana, R. & Kapoor, A. Active learning with model selection. In *Proceedings of the AAAI Conference on Artificial Intelligence*, vol. 28 (2014).

[16] Gardner J (2015). Bayesian active model selection with an application to automated audiometry. Adv. Neural Inf. Process. Syst..

[17] Hukushima K, Nemoto K (1996). Exchange Monte Carlo method and application to spin glass simulations. J. Phys. Soc. Jpn..

[18] Nagata K, Sugita S, Okada M (2012). Bayesian spectral deconvolution with the exchange Monte Carlo method. Neural Netw..

[19] Pronzato L, Pázman A (2013). Design of Experiments in Nonlinear Models: Asymptotic Normality, Optimality Criteria and Small-Sample Properties.

[20] Nagata K, Muraoka R, Mototake Y-I, Sasaki T, Okada M (2019). Bayesian spectral deconvolution based on Poisson distribution: Bayesian measurement and virtual measurement analytics (VMA). J. Phys. Soc. Jpn..

[21] Gelman A, Carlin JB, Stern HS, Rubin DB (1995). Bayesian Data Analysis.

[22] Kass RE, Raftery AE (1995). Bayes factors. J. Am. Stat. Assoc..

[23] Mototake Y-I, Mizumaki M, Akai I, Okada M (2019). Bayesian Hamiltonian selection in X-ray photoelectron spectroscopy. J. Phys. Soc. Jpn..

[24] Cappé O, Godsill SJ, Moulines E (2007). An overview of existing methods and recent advances in sequential Monte Carlo. Proc. IEEE.

[25] Kashiwamura S (2022). Bayesian spectral deconvolution of X-ray absorption near edge structure discriminating between high-and low-energy domains. J. Phys. Soc. Jpn..

[26] Hoffman MD, Gelman A (2014). The No-U-turn sampler: Adaptively setting path lengths in Hamiltonian Monte Carlo. J. Mach. Learn. Res..

[27] Tokuda S (2021). Unveiling quasiparticle dynamics of topological insulators through Bayesian modelling. Commun. Phys..

