# Supplementary material for: Bayesian active learning with model selection for spectral experiments
Source: Sci Rep. 2024 Feb 14;14:3680. doi: 10.1038/s41598-024-54329-w (PMC10866988; doi:10.1038/s41598-024-54329-w)
Supplement: Supplementary file 1 — Supplementary Information. [file 41598_2024_54329_MOESM1_ESM.pdf]

Supplementary Materials: Bayesian Active Learning with Model Selection for Spectral Experiments

Tomohiro Nabika[1], Kenji Nagata[2], Masaichiro Mizumaki[3], Shun Katakami[1], and Masato Okada[1,*]

[1]Graduate School of Frontier Sciences, The University of Tokyo, Kashiwa, Chiba 277-8561, Japan
[2]Research and Services Division of Materials Data and Integrated System, National Institute for Materials Science,Tsukuba, Ibaraki 305-0047, Japan
[3]Faculty of Science, Course for Physical Sciences, Kumamoto University, Japan
[*]Corresponding author: Masato Okada, okada@edu.k.u-tokyo.ac.jp

1 Detailed derivation of two criteria

In this section, we provide the detailed derivation of the two criteria $\widetilde{\mathcal{I}}_M(x)$ and $\widetilde{\mathcal{I}}_{s,c}(x)$.

1.1 Derivation of $\widetilde{\mathcal{I}}_M(x)$

$\mathcal{I}_M(x)$ is bounded by $\widetilde{\mathcal{I}}_M(x)$. First, we transform $\mathcal{I}_M(x)$ as follows:

$$\mathcal{I}_M(x) = \int \mathcal{J}_M(x;y)p(y|x,D,M)\mathrm{d}y \tag{1}$$
$$= \int \{H(p(\theta_M|D,M)) - H(p(\theta_M|D\cup\{x,y\},M))\}\, p(y|x,D,M)\mathrm{d}y \tag{2}$$
$$= \int\int p(\theta_M|D\cup\{x,y\},M)\log(p(\theta_M|D\cup\{x,y\},M))p(y|x,D,M)\mathrm{d}\theta_M\mathrm{d}y \tag{3}$$
$$-\int p(\theta_M|D,M)\log(p(\theta_M|D,M))\mathrm{d}\theta_M \tag{4}$$
$$= \int\int p(\theta_M|D\cup\{x,y\},M)\log(p(\theta_M|D\cup\{x,y\},M))p(y|x,D,M)\mathrm{d}\theta_M\mathrm{d}y \tag{5}$$
$$-\int\int p(\theta_M|D\cup\{x,y\},M)p(y|x,D,M)\log(p(\theta_M|D,M))\mathrm{d}\theta_M\mathrm{d}y \tag{6}$$
$$= \int\int p(\theta_M|D\cup\{x,y\},M)p(y|x,D,M)\log\left(\frac{p(\theta_M|D\cup\{x,y\},M)p(y|x,D,M)}{p(\theta_M|D,M)p(y|x,D,M)}\right)\mathrm{d}\theta_M\mathrm{d}y \tag{7}$$
$$= \int\int p(\theta_M|D,M)p(y|x,\theta_M,M)\log\left(\frac{p(y|x,\theta_M,M)}{p(y|x,D,M)}\right)\mathrm{d}\theta_M\mathrm{d}y \tag{8}$$
$$= \int_\Theta \mathrm{KL}(p_{x,\theta_M}||p_{x,D})p_D(\theta_M)\mathrm{d}\theta_M, \tag{9}$$

where $p_{x,\theta_M}(y) = p(y|x,\theta_M,M)$, $p_D(\theta_M) = p(\theta_M|D,M)$, $p_{x,D}(y) = p(y|x,D,M) = \int p(y|x,\theta_M,M)p_D(\theta_M)\mathrm{d}\theta$, and $\mathrm{KL}(p||q)$ is the Kullback-Leibler (KL) divergence between p and q. From the convexity of KL divergence, $\mathcal{I}_M(x)$ is

bounded as follows:

$$
\begin{aligned}
&\mathcal{I}_M(x) \\
&= \int_\Theta \mathrm{KL}\left(p_{x,\theta_M} \middle\| \int p_{x,\theta'_M} p_D(\theta_M)\mathrm{d}\theta'_M\right) p_D(\theta_M)\mathrm{d}\theta_M, && (10)\\
&\leq \int\int \mathrm{KL}(p_{x,\theta_M}||p_{x,\theta'_M}) p_D(\theta_M) p_D(\theta'_M)\mathrm{d}\theta'_M \mathrm{d}\theta_M && (11)\\
&= \widetilde{\mathcal{I}}_M(x) && (12)
\end{aligned}
$$

1.2 Derivation of $\widetilde{\mathcal{I}}_{s,c}(x)$

$\mathcal{I}_{s,c}(x)$ is bounded by $\widetilde{\mathcal{I}}_{s,c}(x)$. First, we transform $\mathcal{I}_{s,c}(x)$ as follows:

$$
\begin{aligned}
\mathcal{I}_{s,c}(x) &= \int \log \frac{p(M_c|D \cup \{x,y\})}{p(M_s|D \cup \{x,y\})} p(y|x,D)\mathrm{d}y && (13)\\
&= \int \log \frac{p(y|x,D,M_c)p(M_c|D)}{p(y|x,D,M_s)p(M_s|D)} p(y|x,D,M_c)\mathrm{d}y && (14)\\
&= \int \log \frac{p(y|x,D,M_c)}{p(y|x,D,M_s)} p(y|x,D,M_c)\mathrm{d}y + C. && (15)
\end{aligned}
$$

where C is a constant independent of x. From the convexity of KL divergence, $\mathcal{I}_{s,c}(x)$ is bounded as follows:

$$
\begin{aligned}
&\mathcal{I}_{s,c}(x) - C \\
&= \mathrm{KL}\left(\int p_{x,\theta_{M_c}} p_D(\theta_{M_c})\mathrm{d}\theta_{M_c} \middle\| \int p_{x,\theta_{M_s}} p_D(\theta_{M_s})\mathrm{d}\theta_{M_s}\right) && (16)\\
&\leq \int\int \mathrm{KL}(p_{x,\theta_{M_c}}||p_{x,\theta_{M_s}}) p_D(\theta_{M_c}) p_D(\theta_{M_s})\mathrm{d}\theta_{M_c}\mathrm{d}\theta_{M_s} && (17)\\
&= \widetilde{\mathcal{I}}_{s,c}(x) && (18)
\end{aligned}
$$

2 Algorithm for active learning with GPR

The detailed algorithm for active learning with GPR has been provided in this section. Let us assume that for an input $x \in \mathcal{X}$, the response, y, can be written as $y = f(x) + \varepsilon$ ($\varepsilon \sim \mathcal{N}(0, \xi^2)$) using the modeling function f, which follows the Gaussian process. For the input, $x_1, ..., x_s \in \mathcal{X}$, $f(x_1), ..., f(x_s)$ follows a Gaussian distribution with mean $\mu\mathbf{1}$, where $\mu = \frac{\sum_{i=1}^{s} y_i}{s}$, and the covariance matrix $\boldsymbol{K} = \{k(x_n, x_{n'})\}_{n,n'}$, where the kernel function $k(x, x')$ is a Gaussian kernel given by

$$
k(x, x') = \theta_1 \exp\left(-\left(\frac{x - x'}{\theta_2}\right)^2\right). \tag{19}
$$

Given data $D = \{(x_1, y_1), ..., (x_N, y_N)\}$, the mean $\grave{\mu}(x)$ and the covariance $\grave{\sigma}^2(x)$ of the prior distribution of $f(x)$ is given as follows [1]:

$$
\begin{aligned}
\grave{\mu}(x) &= \mu_0 + \boldsymbol{k}_N(x)^\top (\boldsymbol{K} + \xi^2 \boldsymbol{I}_n)^{-1}(\boldsymbol{y} - \mu_0\mathbf{1}), && (20)\\
\grave{\sigma}^2(x) &= k(x,x) - \boldsymbol{k}_N(x)^\top (\boldsymbol{K} + \xi^2 \boldsymbol{I}_N)^{-1}\boldsymbol{k}_N(x), && (21)
\end{aligned}
$$

where $\mu_0 = \frac{\sum_{i=1}^{N} y_i}{N}$ and $\boldsymbol{k}_N(x) = (k(x_1, x), ..., k(x_N, x))$. Here, hyperparameters θ_1, θ_2, and ξ are determined to maximize the likelihood $p(\boldsymbol{y}|\boldsymbol{x}, \theta_1, \theta_2, \xi)$, where $\boldsymbol{x} = (x_1, ..., x_N)$ and $\boldsymbol{y} = (y_1, ..., y_N)$. We use $\grave{\sigma}^2(x)$ for the criterion of active learning, i.e., we select n measurement points from $x \in \mathcal{X}$ with a large $\grave{\sigma}^2(x)$ as the next measurement points. The detailed algorithm are shown in Algorithm 1. To reduce the computational complexity of Gaussian process regression, when the number of data points exceeds 1000, a random selection of 1000 data points was made for training. We implement the Gaussian process using the GPy [2].

Algorithm 1 Active learning with GPR

Require: Number of measurement points per one experiment n, Number of experiments k, Measurement points set $\mathcal{X} = \{x_i\}_{i=1}^N$
Ensure: Data $D = \{(x_i, y_i/t_i, t_i)\}_{i=1}^N$
Measure $y_1, ..., y_N$ with $x_1, ..., x_N$.
Data $D = \{(x_i, y_i/T, T)\}_{i\in\{1,...,N\}}$
for $i \in \{1, ..., k\}$ **do**
    Calculate criteria $\{\grave{\sigma}^2(x_i)\}_{x_i\in\mathcal{X}}$.
    Select n points $\{x'_1, ..., x'_n\}$ in descending order of $\{\grave{\sigma}^2(x_i)\}_{x_i\in\mathcal{X}}$.
    Measure $\{y'_1, ..., y'_n\}$ in $\{x'_1, ..., x'_n\}$.
    for $x_j \in \{x'_1, ..., x'_n\}$ **do**
        $y_j = (y_j \times t_j + y'_j \times T)/(t_j + T)$
        $t_j = t_j + T$
    end for
end for

3 Experiment details

3.1 Spectral deconvolution

Prior distribution

Let $\eta_a = 2.0, \lambda_a = 1.0,\ \nu_0 = 157.0, \xi_0 = 167.0, \eta_\sigma = 10.0, \lambda_\sigma = 2.5,\ \nu_B = 0.1, \xi_B = 0.01$, and we set the prior distributions of $\{a_k, \mu_k, \sigma_k\}_{k=1}^K, B$ be set as follows:

$$\varphi(a_k) = \mathrm{Gamma}\left(a_k; \eta_a, \lambda_a\right) \tag{22}$$

$$= \frac{1}{\Gamma(\eta_a)}(\lambda_a)^{\eta_a}(a_k)^{\eta_a-1}\exp\left(-\lambda_a a_k\right) \tag{23}$$

$$\varphi(\mu_k) = U(\nu_0, \nu_1) \tag{24}$$

$$\varphi(\sigma_k) = \mathrm{Gamma}\left(\frac{1}{\sigma_k^2}; \eta_\sigma, \lambda_\sigma\right) \tag{25}$$

$$\varphi(B) = N(B; \nu_B, {\xi_B}^2), \tag{26}$$

$$\tag{27}$$

where $\Gamma(\eta_a)$ is the gamma function, $U(\nu_0, \nu_1)$ is the uniform distribution on $[\nu_0, \nu_1]$, and $N(B; \nu_B, {\xi_B}^2)$ is the Gaussian distribution of mean ν_B and variance ${\xi_B}^2$.

Let the prior distribution of the model set be $\varphi(M_k) = \frac{1}{|\mathcal{M}|}$.

Hyperparameters for the exchange Monte Carlo method

Regarding the hyperparameter of EMC, we set the replica size $L = 40$, and the inverse temperature $\{\beta_l\}_{l=1}^L$ as follows:

$$\beta_l = \begin{cases} 0 & (l = 1), \\ 1.4^{l-L} & (\text{otherwise}). \end{cases} \tag{28}$$

We set the burn in size $T_1 = 5000$, the sample size $T_2 = 5000$ for an active learning and the burn in size $T_1 = 20000$, the sample size $T_2 = 20000$ for the final estimation.

3.2 Hamiltonian selection

3.2.1 Hamiltonian model

Let ϵ_L, ϵ_f^0 and ϵ_c be the energies of the conducting electrons of 4f rare-earth metals (5d, 6s electrons), the 4f electron, and the core electron, respectively. We set the index ν ($\nu = 1, ..., N_f, N_f = 14$) as the quantum number of the

spin and f orbital. Moreover, let V, U_{ff} and $-U_{fc}$ be the energies of the hybridization interaction between the 4f electrons and the conduction electrons, the Coulomb interaction between the 4f electrons, and the core-hole Coulomb potential for the 4f electrons, respectively. We define $\Delta = \epsilon_f^0 - \epsilon_L$. Let M_2 be a model using a two-state Hamiltonian H_2 and M_3 be a model using a three-state Hamiltonian H_3, and let $\mathcal{M} = \{M_2, M_3\}$ be the set of candidate models.

The two-state Hamiltonian H_2 is the effective Hamiltonian for the XPS spectrum of La_2O_3 and was proposed by [3]. The Hamiltonian is given by

$$H_2 = \epsilon_L \sum_{\nu=1}^{N_f} a_{L\nu}^\dagger a_{L\nu} + \epsilon_f^0 \sum_{\nu=1}^{N_f} a_{f\nu}^\dagger a_{f\nu} + \epsilon_c a_c^\dagger a_c + \frac{V}{\sqrt{N_f}} \sum_{\nu=1}^{N_f} (a_{L\nu}^\dagger a_{f\nu} + a_{L\nu} a_{f\nu}^\dagger) - U_{fc} \sum_{\nu=1}^{N_f} a_{f\nu}^\dagger a_{f\nu} (1 - a_c^\dagger a_c), \tag{29}$$

where $|G\rangle$ is the eigenstate of the minimum energy E_G in the initial state and $|F_j\rangle\,(j = 0, 1)$ is the eigenstate of the two energy levels $E_j (j = 0, 1)$ in the final state. To compare the two models, we introduce the energy shift parameter (b). Here, we set the parameter $\theta_{M_2} = \{\Delta, V, \Gamma, U_{fc}, b\}$ and the modeling function as

$$f_{M_2}(x; \theta_{M_2}) = \sum_{j=0}^{1} |\langle F_j | a_c |G\rangle|^2 \frac{\Gamma/\pi}{(x - (E_j - E_g) - b)^2 + \Gamma^2}. \tag{30}$$

Specifically, to obtain the modeling function $f_{M_2}(x; \theta_{M_2})$, we calculate

$$f_{M_2}(x; \theta_{M_2}) = \sum_{j=0}^{1} |(\boldsymbol{f}_j, \boldsymbol{g})|^2 \frac{\Gamma/\pi}{(x - (E_j - E_g) - b)^2 + \Gamma^2} \tag{31}$$

where E_g, $\boldsymbol{g}$ are the minimum eigenvalue and its eigenvector of $\begin{pmatrix} 0 & \sqrt{N_f}V \\ \sqrt{N_f}V & \Delta - U_{fc} \end{pmatrix}$ respectively, and E_0, E_1 and $\boldsymbol{f}_0, \boldsymbol{f}_1$ are the eigenvalues and their eigenvectors of $\begin{pmatrix} 0 & \sqrt{N_f}V \\ \sqrt{N_f}V & \Delta - U_{fc} \end{pmatrix}$.

The three-state Hamiltonian H_3 is the effective Hamiltonian for the XPS spectrum of CeO_2 and was proposed by [4]. The Hamiltonian is given by

$$H_3 = \epsilon_L \sum_{\nu=1}^{N_f} a_{L\nu}^\dagger a_{L\nu} + \epsilon_f^0 \sum_{\nu=1}^{N_f} a_{f\nu}^\dagger a_{f\nu} + \epsilon_c a_c^\dagger a_c + \frac{V}{\sqrt{N_f}} \sum_{\nu=1}^{N_f} (a_{L\nu}^\dagger a_{f\nu} + a_{L\nu} a_{f\nu}^\dagger) \tag{32}$$

$$+ U_{ff} \sum_{\nu>\nu'} a_{f\nu}^\dagger a_{f\nu} a_{f\nu'}^\dagger a_{f\nu'} - U_{fc} \sum_{\nu=1}^{N_f} a_{f\nu}^\dagger a_{f\nu} (1 - a_c^\dagger a_c), \tag{33}$$

where $|G\rangle$ is the eigenstate of the minimum energy E_G in the initial state and $|F_j\rangle\,(j = 0, 1, 2)$ is the eigenstate of the three energy levels $E_j (j = 0, 1, 2)$ in the final state. As in the case of H_2, we introduce the energy shift parameter b. Here, we set the parameter $\theta_{M_3} = \{\Delta, V, \Gamma, U_{fc}, U_{ff}, b\}$ and the modeling function as follows:

$$f_{M_3}(x; \theta_{M_3}) = \sum_{j=0}^{2} |\langle F_j | a_c |G\rangle|^2 \frac{\Gamma/\pi}{(x - (E_j - E_g) - b)^2 + \Gamma^2}. \tag{34}$$

Specifically, to obtain the modeling function $f_{M_3}(x; \theta_{M_3})$, we calculate

$$f_{M_3}(x; \theta_{M_3}) = \sum_{j=0}^{2} |(\boldsymbol{f}_j, \boldsymbol{g})|^2 \frac{\Gamma/\pi}{(x - (E_j - E_g) - b)^2 + \Gamma^2}, \tag{35}$$

where E_g, $\boldsymbol{g}$ are the minimum eigenvalue and its eigenvector of $\begin{pmatrix} 0 & \sqrt{N_f}V & 0 \\ \sqrt{N_f}V & \Delta & \sqrt{2(N_f-1)}V \\ 0 & \sqrt{2(N_f-1)}V & 2\Delta + U_{ff} \end{pmatrix}$ respectively, and E_0, E_1, E_2 and $\boldsymbol{f}_0, \boldsymbol{f}_1, \boldsymbol{f}_2$ are the eigenvalues and their eigenvectors of $\begin{pmatrix} 0 & \sqrt{N_f}V & 0 \\ \sqrt{N_f}V & \Delta & \sqrt{2(N_f-1)}V \\ 0 & \sqrt{2(N_f-1)}V & 2\Delta - 2U_{fc} + U_{ff} \end{pmatrix}$.

Prior distribution

We set the prior distribution of the parameters as follows:

$$\varphi(\Delta) = U(0, 20), \tag{36}$$
$$\varphi(V) = U(0, 4), \tag{37}$$
$$\varphi(U_{ff}) = U(0, 20), \tag{38}$$
$$\varphi(U_{fc}) = U(0, 20), \tag{39}$$
$$\varphi(\Gamma) = U(0.01, 1), \tag{40}$$
$$\varphi(b) = U(-5.0, 5.0). \tag{41}$$

Moreover, the prior distribution of the model is set as $\varphi(M_2) = \varphi(M_3) = \frac{1}{2}$.

Hyperparameters for the exchange Monte Carlo method

Regarding the hyperparameter of EMC, we set the replica size $L = 40$, and the inverse temperature $\{\beta_l\}_{l=1}^{L}$ as follows:

$$\beta_l = \begin{cases} 0 & (l = 1), \\ 1.3^{l-L} & (\text{otherwise}). \end{cases} \tag{42}$$

We set the burn in size $T_1 = 5000$, the sample size $T_2 = 5000$ for an active learning and the burn in size $T_1 = 50000$, the sample size $T_2 = 50000$ for the final estimation.

References

[1] Ueno, T., Ishibashi, H., Hino, H. & Ono, K. Automated stopping criterion for spectral measurements with active learning. *npj Computational Materials* **7**, 139 (2021).

[2] GPy. Gpy: A Gaussian process framework in python (2012). URL `http://github.com/SheffieldML/GPy`.

[3] Kotani, A. & Toyozawa, Y. Photoelectron spectra of core electrons in metals with an incomplete shell. *Journal of the Physical Society of Japan* **37**, 912–919 (1974).

[4] Kotani, A., Mizuta, H., Jo, T. & Parlebas, J. Theory of core photoemission spectra in CeO_2. *Solid state communications* **53**, 805–810 (1985).
